# Angiotensin II‐induced redox‐sensitive SGLT1 and 2 expression promotes high glucose‐induced endothelial cell senescence

**DOI:** 10.1111/jcmm.14233

**Published:** 2019-03-30

**Authors:** Sonia Khemais‐Benkhiat, Eugenia Belcastro, Noureddine Idris‐Khodja, Sin‐Hee Park, Lamia Amoura, Malak Abbas, Cyril Auger, Laurence Kessler, Eric Mayoux, Florence Toti, Valérie B. Schini‐Kerth

**Affiliations:** ^1^ UMR CNRS 7213 Laboratoire de Biophotonique et Pharmacologie, Faculté de Pharmacie Université de Strasbourg Illkirch France; ^2^ UMR INSERM 1109 Nanomédecine Régénérative Ostéo‐articulaire et Dentaire, Faculté de Médecine FMTS Université de Strasbourg Strasbourg France; ^3^ EA7293 Stress Vasculaire et Tissulaire en Transplantation Faculté de Pharmacie FMTS Université de Strasbourg Illkirch France; ^4^ Boehringer Ingelheim Pharma GmbH & Co. KG Biberach Germany

**Keywords:** angiotensin system, endothelial cells, high glucose, pro‐atherothrombotic markers, senescence, SGLT2

## Abstract

High glucose (HG)‐induced endothelial senescence and dysfunction contribute to the increased cardiovascular risk in diabetes. Empagliflozin, a selective sodium glucose co‐transporter2 (SGLT2) inhibitor, reduced the risk of cardiovascular mortality in type 2 diabetic patients but the protective mechanism remains unclear. This study examines the role of SGLT2 in HG‐induced endothelial senescence and dysfunction. Porcine coronary artery cultured endothelial cells (ECs) or segments were exposed to HG (25 mmol/L) before determination of senescence‐associated beta‐galactosidase activity, protein level by Western blot and immunofluorescence staining, mRNA by RT‐PCR, nitric oxide (NO) by electron paramagnetic resonance, oxidative stress using dihydroethidium and glucose uptake using 2‐NBD‐glucose. HG increased ECs senescence markers and oxidative stress, down‐regulated eNOS expression and NO formation, and induced the expression of VCAM‐1, tissue factor, and the local angiotensin system, all these effects were prevented by empagliflozin. Empagliflozin and LX‐4211 (dual SGLT1/2 inhibitor) reduced glucose uptake stimulated by HG and H_2_O_2_ in ECs. HG increased SGLT1 and 2 protein levels in cultured ECs and native endothelium. Inhibition of the angiotensin system prevented HG‐induced ECs senescence and SGLT1 and 2 expression. Thus, HG‐induced ECs ageing is driven by the local angiotensin system via the redox‐sensitive up‐regulation of SGLT1 and 2, and, in turn, enhanced glucotoxicity.

## 1 INTRODUCTION

Diabetes mellitus is a major public health problem currently affecting more than 387 million people worldwide and this number is estimated to increase to 592 million by 2035.^1^ Both microvascular complications, including retinopathy, nephropathy and neuropathy and macrovascular complications including ischaemic heart diseases and cerebrovascular diseases increase the risk of premature death from cardiovascular disease, the leading cause of death, in diabetes mellitus.^2^ Endothelial dysfunction is an early key event contributing to the initiation and development of diabetic vascular complications.^3^, ^4^ Indeed, reduced endothelium‐dependent nitric oxide (NO)‐mediated vasodilatation is observed in patients with type 1 and 2 diabetes,^5^, ^6^, ^7^ and blunted NO‐mediated vasorelaxation in experimental models of diabetes.^8^, ^9^, ^10^ Since exposure of isolated arteries and cultured endothelial cells (ECs) to high glucose (HG) reduced the formation of NO,^11^, ^12^, ^13^ ECs appear to be particularly sensitive to glucotoxicity. The mechanisms underlying the hyperglycemia‐induced endothelial dysfunction have been shown to involve NADPH oxidase‐ and mitochondria‐derived oxidative stress,^10^, ^14^ uncoupled endothelial NO synthase,^10^ advanced glycation end products (AGEs) and receptors for AGEs signalling,^15^ and increased levels of asymmetric dimethylarginine.^16^ More recently, endothelial senescence has been identified as a potential contributor to the premature alteration of the endothelial function in diabetes. Indeed, senescent ECs were observed in the aorta of diabetic rats,^17^, ^18^, ^19^ and the exposure of ECs to a high concentration of glucose (HG) increased the number of senescence associated‐beta‐galactosidase (SA‐β‐gal) positive cells.^18^, ^19^ Endothelial senescence, characterized by an irreversible form of growth arrest, is associated with functional and structural changes and altered gene expression including increased oxidative stress and the down‐regulation of the eNOS‐derived formation of NO, and, as a consequence, an up‐regulation of pro‐atherothrombotic responses.^18^, ^19^

Gliflozins are a new class of anti‐diabetic drugs targeting the sodium‐glucose co‐transporter 2 (SGLT2), located in the S1‐S2 segments of the proximal tubule in the kidney and responsible for nearly 90% of renal glucose reabsorption. In addition to SGLT2, SGLT1 in the distal segment S3 accounts for the reabsorption of the remaining 10%.^20^ Thus, inhibition of SGLT2 leads to a significant reduction in the blood glucose level by preventing glucose reabsorption by the kidney. Empagliflozin, a selective SGLT2 inhibitor, reduced cardiovascular mortality in type 2 diabetic patients by 38%, in parallel to a moderate reduction in HbA1c,^21^ and decreased the progression of kidney disease and lowered the rate of clinically relevant renal events.^22^ The role of SGLT2 in diabetes‐related endothelial dysfunction and vascular complications has been poorly studied. Recently, SGLT inhibitors have been shown to attenuate the endothelial dysfunction in streptozotocin‐treated mice and rats 23, 24 and in Zucker diabetic fatty rats.^20^ However, it is unclear whether the vasoprotective effect of SGLT inhibitors is related to the decreased renal sodium and glucose reabsorption and/or to a direct effect at the arterial wall. Indeed, SGLT1 expression has been observed in cultured ECs and in the endothelium of the mouse thoracic aorta, and its expression is increased in capillaries and small vessels after brain ischemia and reperfusion.^25^, ^26^ In contrast to SGLT1, studies regarding the expression of SGLT2 in ECs have indicated that SGLT2 mRNA levels were undetectable in control cultured human pulmonary and coronary artery EC lines.^27^ Since cultured ECs often undergo pronounced phenotypic changes following numerous passages, SGLT2 expression remains to be determined in primary and low passages of ECs under both physiological and pathological conditions. Therefore, the aim of this study was to investigate the possibility that SGLT2 may play a role in endothelial senescence and dysfunction following chronic exposure to HG raising the interest and the understanding of the use of SGLT2 inhibitors in type 2 diabetes.

## 2 MATERIALS AND METHODS

### 2.1 Preparation of coronary artery rings and cultured endothelial cells

Porcine hearts were collected from the local slaughterhouse (SOCOPA, Holtzheim, France). Left circumflex coronary arteries were excised, cleaned and flushed with PBS without calcium to remove remaining blood. For immunofluorescence staining, coronary artery segments with endothelium were incubated in RPMI containing a normal glucose concentration (NG: 11.1 mmol/L) supplemented with fungizone (2.5 μg/mL), penicillin (100 U/mL), streptomycin (100 μg/mL), HEPES (100 mmol/L), in the absence or presence of empagliflozin (100 nmol/L) or LX‐4211 (10 nmol/L) for 30 minutes before the subsequent incubation in normo glucose or high glucose (HG: 25 mmol/L) for 24 hours. Thereafter, segments were washed with PBS before being embedded in FSC22 Frozen section medium (Leica Biosystem, France) and frozen in liquid nitrogen. To prepare primary cultures, ECs were isolated by collagenase treatment (Type I, ThermoFisher, 1 mg/mL for 12 min at 37°C), and cultured in culture dishes containing MCDB 131 medium (Invitrogen, LifeTechnologies SAS, Courtaboeuf, France) supplemented with fungizone (2.5 μg/mL), penicillin (100 U/mL), streptomycin (100 μg/mL), L‐glutamine (2 mmol/L, all from Lonza, Levallois‐Perret, France) and 15% foetal calf serum, and grown for 48‐72 hours (passage 0). To induce premature senescence, ECs at passage 1 were incubated with either MCDB 131 with a normal glucose (NG: 5 mmol/L) or a high glucose concentration (HG: 25 mmol/L) for either 48 or 96 h. To induce replicative senescence, ECs were detached with trypsin‐ethylenediaminetetraacetic acid (trypsin‐EDTA; Life Technologies SAS) and further passaged at a ratio of 1:3 at regular intervals. For platelet aggregation experiments, ECs were cultured on Cytodex‐3 beads, which were hydrated and sterilized according to the instructions supplied by the manufacturer (GE Healthcare Life Sciences, Strasbourg, France). ECs at passage 1 grown on Cytodex‐3 beads were incubated in the absence or presence of HG for 96 h before their addition to platelet suspensions.^28^

### 2.2 Determination of SA‐β‐gal activity by flow cytometry

SA‐β‐galactosidase activity was determined by flow cytometry using the fluorogenic substrate C_12_FDG (5‐dodecanoylaminofluorescein Di‐β‐D‐galactopyranoside, Invitrogen) as described previously.^29^ ECs were pretreated with 300 μmol/L chloroquine for 1 hour to induce lysosomal alkalinization. C_12_FDG (33 μmol/L) was then added to the incubation medium for 1 hour. At the end of the incubation period, ECs were washed with ice‐cold PBS, resuspended following trypsinization and analysed using a flow cytometer (FACScan, BD Bioscience, Le Pont de Claix, France). Data were acquired and analysed using the Cellquest software (BD Bioscience). Light scatter parameters were used to eliminate dead cells and subcellular debris. The C_12_‐fluorescein signal was measured on the FL1 detector, and the proportion of ECs with SA‐β‐gal activity was estimated using the median fluorescence intensity of the population. Autofluorescence assessed in parallel in ECs not exposed to C_12_FDG was negligible.

### 2.3 Western blot analysis

ECs were washed with PBS and then lysed in extraction buffer (composition in mmol/L: NaCl 150, Na_3_VO_4_ 1, sodium pyrophosphate 10, NaF 20, okadaic acid 0.01 (Sigma, Saint‐Quentin Fallavier, France), Tris/HCl 20 (pH 7.5; QBiogene), a tablet of protease inhibitor (Complete^®^, Roche) and 1% Triton X‐100 (QBiogen, MP Biomedicals, Illkirch, France). Equal amounts of proteins were separated on denaturing SDS (10%‐12%) polyacrylamide gel. Separated proteins were transferred electrophoretically onto nitrocellulose membrane (GE Healthcare Life Sciences). Blots were blocked at room temperature with 5% bovine serum albumin in PBS plus 0.1% Tween 20 (Sigma). For detection of proteins, membranes were incubated with the respective primary antibody: rabbit polyclonal anti‐eNOS (diluted 1:1000; Cell Signaling Technology; Cat. No. 9572), rabbit polyclonal anti‐p53 (diluted 1:1000; Santa Cruz; Cat. No. SC‐6243), mouse monoclonal anti‐p21 (diluted 1:500; Santa Cruz; Cat. No. SC‐271532), mouse monoclonal anti‐p16 (diluted 1:500; Santa Cruz; Cat. No. SC‐390485), rabbit polyclonal anti‐tissue factor (TF, diluted 1:1000; Abcam; Cat. No. ab104513), rabbit polyclonal anti‐VCAM‐1 (diluted 1:1000; Abcam; Cat. No. ab134047), mouse monoclonal anti‐p22^phox^ (diluted 1:1000; Santa Cruz; Cat. No. SC‐5827), rabbit polyclonal anti‐p47^phox^ (diluted 1:1000; Santa Cruz; Cat. No. SC‐14015), mouse monoclonal anti‐cyclooxygenase‐1 (COX‐1, diluted 1:1000; Santa Cruz; Cat. No. SC‐19998), mouse monoclonal anti‐COX‐2 (diluted 1:500; Santa Cruz; Cat. No. SC‐376861), rabbit polyclonal anti‐angiotensin‐converting enzyme (ACE, diluted 1:1000; Abbiotec), rabbit polyclonal anti‐angiotensin type 1 receptor (AT1R, diluted 1:1000; Santa Cruz Technology), rabbit polyclonal anti‐SGLT1 (diluted 1:1000; Santa Cruz; Cat. No. SC‐98974), rabbit polyclonal anti‐SGLT2 (diluted 1:1000; Santa Cruz; Cat. No. SC‐98975) or mouse monoclonal anti‐β‐tubulin (diluted 1:10000; Sigma‐Aldrich; Cat. No. T7816) overnight at 4°C. After washing, membranes were incubated with the secondary antibody (peroxidase‐labeled anti‐rabbit or antimouse immunoglobulin G, dilution of 1:5000; Cell Signaling Technology; Cat. No. 7074, 7076, respectively) at room temperature for 60 minutes. Prestained markers (Euromedex, Souffelweyersheim, France) were used for molecular mass determinations. Immunocomplexes were detected by chemiluminescence reaction (Clarity Western, Biorad, France) followed by densitometric analysis using the software Image J (Image J 1.49p, National Institute of Health, USA).

### 2.4 Analysis of mRNA expression by RT‐PCR

Total RNAs were isolated from ECs or tissues using the mirVANA^®^ Isolation kit (Invitrogen). In some experiments, mRNAs were purified from the total RNAs using a poly (dt) primer, in accordance with the manufacturer's instructions (Macherey‐Nagel, Hoerdt, France). A rat intestine sample was used as positive control for SGLT1, and porcine kidney sample as positive control for SGLT2. cDNA was obtained by reverse transcription of DNase‐free RNA templates using QuantiTect^®^ reverse transcription kit (Quiagen). Primer sequences included porcine SGLT1: 5'GTGGGCAGCTCTTCGATTAC 3’ (sense), 5 ‘AACACAGGCGGTAGAGATGC 3’ (antisense); porcine SGLT2: 5 ‘TGAGTGGAATGCTCTGTTCG 3’ (sense), 5'ACGAAGGTCTGCACCGTATC 3 ‘(antisense); Porcine GAPDH: 5 ‘ACCACAGTCCATGCCATCAC 3’ (sense), 5 ‘TCCACCACCCTGTTGCTGTA 3’ (antisense), and rat 40S ribosomal protein S16 5'TCTGGGCAAGGAGAGATTTG 3’ (sense), 5’ CCGCCAAACTTCTTGGATTC 3’ were used as housekeeping gene. qPCR were made in duplicate with a Bio‐Rad Real Time PCR System. Results were normalized with GAPDH and expressed as fold change over control.

### 2.5 Determination of NO formation by electron paramagnetic resonance

Nitric oxide formation was assessed in ECs cultured on Cytodex‐3 beads by electron paramagnetic resonance after formation of Fe(II)NO(DETC)_2_, a paramagnetic diethyldithiocarbamate iron complex with NO, at 77 K in a Dewar flask using a MS100 spectrometer (Magnettech Ltd., Berlin, Germany). The ESR methodology was used as reported previously.^30^ The values are expressed in signal amplitude (arbitrary units).

### 2.6 Determination of platelet aggregation

Washed human platelet suspensions were kindly provided by the Etablissement Français du Sang – Alsace (Strasbourg), and were prepared as previously described.^28^ Suspensions of washed platelets (450 μL, 3.10^8^ platelets/mL) were incubated for 2 minutes in a Chronolog 490 aggregometer (Diagnostica Stago SAS, Asnière sur Seine, France) with continuous stirring at 1000 r.p.m before addition of a submaximal concentration of U46619 (0.07 μmol/L, a thromboxane A_2_ analog) and fibrinogen (1.6 mg/mL). A volume of 10‐20 μL of beads covered with ECs was added to platelet suspensions 2 minutes before the addition of U46619.

### 2.7 Determination of oxidative stress

ECs were seeded into Lab‐Tek^®^ chamber slide for 24 h, then exposed to serum‐free MCDB 131 (Invitrogen) for 6 hours. The redox‐sensitive fluorescent dye dihydroethidium (DHE) was used to evaluate the formation of reactive oxygen species (ROS). ECs were incubated with DHE (5 μmol/L) for 30 minutes at 37°C in a light protected manner. ECs were then washed and mounted in DAKO medium (fluorescence medium, DAKO, Les Ulis, France) and examined under confocal microscope (Leica SP2 UV DM Irbe). Images were analysed using Image J software.

### 2.8 Determination of glucose uptake analysis by flow cytometry

ECs were seeded at a density of 2 × 10^5^ cells per well in a 6 well plate and incubated overnight. Then, ECs were incubated in serum‐free medium for 6 hours before being washed once with PBS and incubated with 100 μmol/L of 2‐(N‐(7‐Nitrobenz‐2‐oxa‐1,3‐diazol‐4‐yl)Amino)‐2‐Deoxyglucose (2‐NBD‐glucose, Life Technologies, SAS) for 1 h at 37°C. Thereafter, ECs were trypsinized before being centrifuged at 300 g for 5 minutes at room temperature and washed once with PBS. Cell pellets were resuspended in 300 μl PBS and the 2‐NBD‐glucose fluorescence was determined in the FITC channel (FL‐1) using a flow cytometer (FACScan). Mean fluorescence intensity of 2‐NBD‐glucose was used to measure glucose uptake by ECs. Unstained control was used to optimize FACS settings.

### 2.9 Immunofluorescence studies

For immunofluorescence histochemistry, 0.14 μm cryomicrotome sections of porcine coronary artery segments were fixed with paraformaldehyde 4% (w/v) for 30 minutes, and then incubated with blocking solution (PBS + BSA 1% (w/v) + Triton X‐100 0.5% (w/v)) for 30 minutes at room temperature. Sections were subsequently incubated with either purified mouse anti‐eNOS/NOS Type III (1:100, Bd Bioscences), rabbit anti‐VCAM‐1 [EPR5047] (1:250, Abcam), rabbit anti‐SGLT‐1 or anti‐SGLT‐2 (1:100, Alomone Labs, Ltd.) primary antibody for 60 minutes at 4°C. After washing with PBS, sections were incubated with 1:500 dilution of a secondary polyclonal goat antimouse immunoglobulin G coupled to CF 633 (Alexa Fluor 633 conjugate, Invitrogen) or a polyclonal goat anti‐rabbit immunoglobulin G coupled to CF 633 (Alexa Fluor 633 conjugate, Invitrogen), respectively for 60 minutes at room temperature in the dark. For negative controls, the primary antibody was omitted. After washing with PBS, sections were incubated with 4, 6‐diamidino‐2 ‐phenylindole, dihydrochloride (DAPI, Thermo Fisher) for 3 minutes at room temperature to counterstain nuclei. Thereafter, sections were washed with PBS and mounted in Dako fluorescence mounting medium (Dako) and cover‐slipped before being evaluated by confocal microscopy using a confocal laser‐scanning microscope (Leica SP2 UV DM Irbe). Quantification of fluorescence levels was performed using Image J software (version 1.49p for Windows, US National Institutes of Health, Bethesda, MD, USA) following subtraction of the autofluorescence.

### 2.10 Statistical Analysis

Data are presented as mean ± SEM of n different experiments. Mean values were compared using Student's paired *t* test or an analysis of variance followed by the post‐hoc Bonferroni test to identify significant differences between treatments using GraphPad Prism (v5.0). The difference was considered to be significant when the *P* value was less than 0.05.

## 3 RESULTS

### 3.1 High glucose‐induced endothelial senescence is prevented by empagliflozin

**Figure 1:**
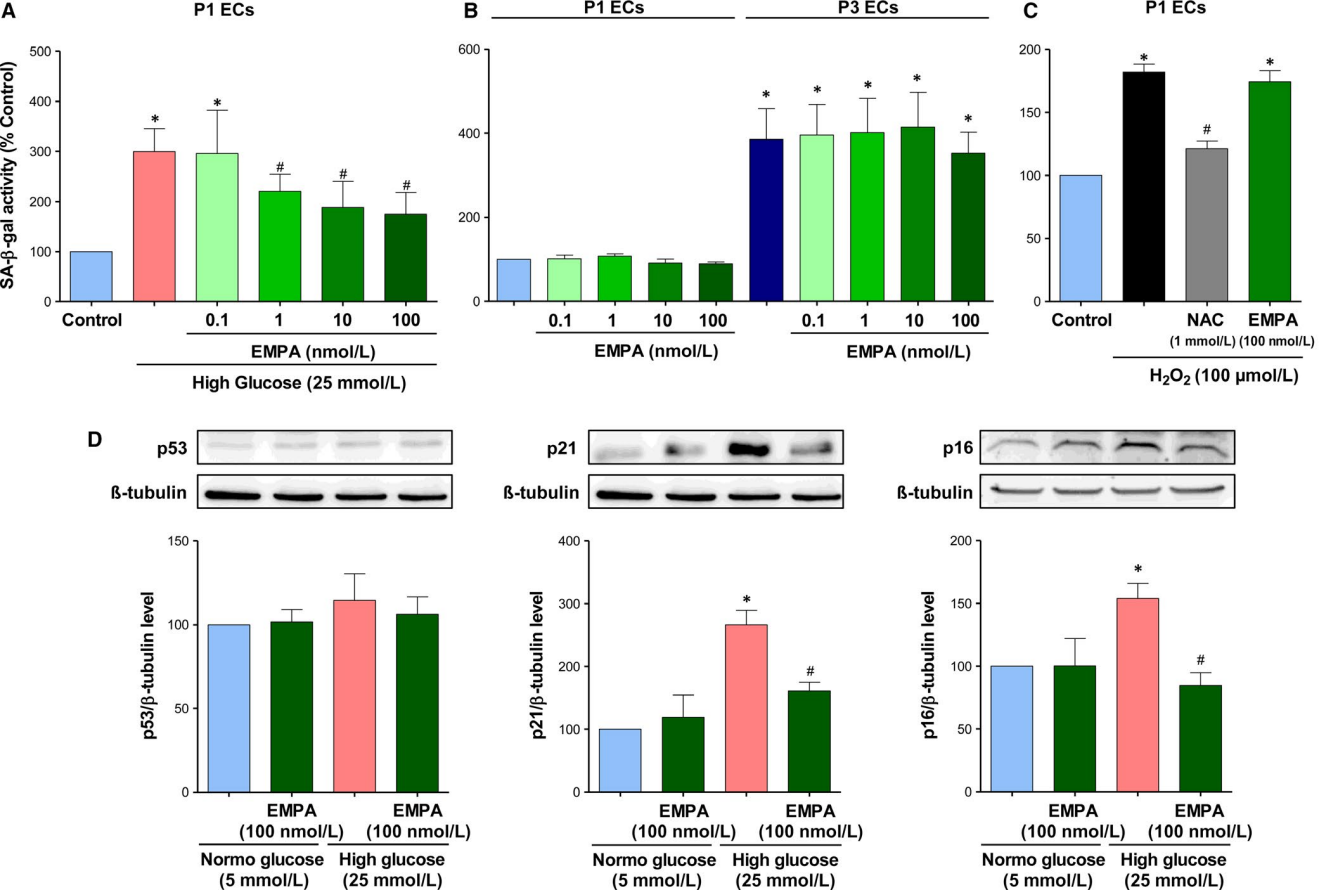
The selective SGLT2 inhibitor empagliflozin prevents the high glucose‐induced ECs senescence but not replicative senescence and H_2_O_2_‐induced senescence. A, ECs at P1 are exposed either to normal (5 mmol/L, NG) or high glucose (25 mmol/L, HG) in the absence or presence of increasing concentrations of empagliflozin for 96 h. B, ECs at P1 or P3 are exposed to increasing concentrations of empagliflozin for 48 h. C, ECs at P1 are exposed to N‐acetyl cysteine (NAC, an antioxidant, 1 mmol/L) or empagliflozin (EMPA, 100 nmol/L) for 30 min before being exposed to 100 μmol/L of H_2_O_2_ for 1 h. Thereafter, the medium is replaced and ECs are incubated for 48 h. SA‐β‐gal activity is determined by flow cytometry. D, ECs are exposed either to normal or high glucose for 96 h in the presence or absence of empagliflozin (100 nmol/L) before Western blot analysis of p53, p21, and p16. Results are shown as representative immunoblots (upper panels) and corresponding cumulative data (lower panels). Data are expressed as mean ± SEM of n = 3‐4. *P <* *0.05 vs. control NG and ^#^ *P *<* *0.05 vs. control HG

The role of SGLT2 in HG‐induced endothelial senescence, an early event promoting endothelial dysfunction,^31^ is examined using the selective SGLT2 inhibitor empagliflozin in cultured porcine coronary artery endothelial cells (ECs) and assessed by the SA‐β‐gal activity using C_12_FDG. Treatment of ECs with empagliflozin inhibited the HG‐induced ECs senescence in a concentration‐dependent manner with a significant effect observed at concentrations within the range of 1‐100 nmol/L (Figure 1A). The inhibitory effect of empagliflozin amounted to about 63% at 100 nmol/L (Figure 1A). In contrast, empagliflozin did not affect the low level of senescence in ECs at P1 and replicative senescence assessed in ECs at P3 (Figure 1B). Since oxidative stress has been involved in HG‐induced ECs senescence,^19^ experiments were performed to determine the possibility that empagliflozin affects oxidative stress‐induced premature ECs senescence via short‐term exposure (1 h) of cells to H_2_O_2_. The H_2_O_2_‐induced increased SA‐β‐gal activity was markedly prevented by the antioxidant N‐acetyl‐cysteine but not by empagliflozin (Figure 1C) indicating that the SGLT2 inhibitor does not affect the signal transduction pathway leading to senescence in response to oxidative stress. Consistent with an increased SA‐β‐gal activity, HG up‐regulated the expression level of the senescence markers p21 and p16 but not p53, and this effect was prevented by empagliflozin (Figure 1D). Empagliflozin alone did not affect the low basal expression level of p53, p21 and p16 (Figure 1D).

### 3.2 Empagliflozin prevents glucotoxicity in ECs

**Figure 2:**
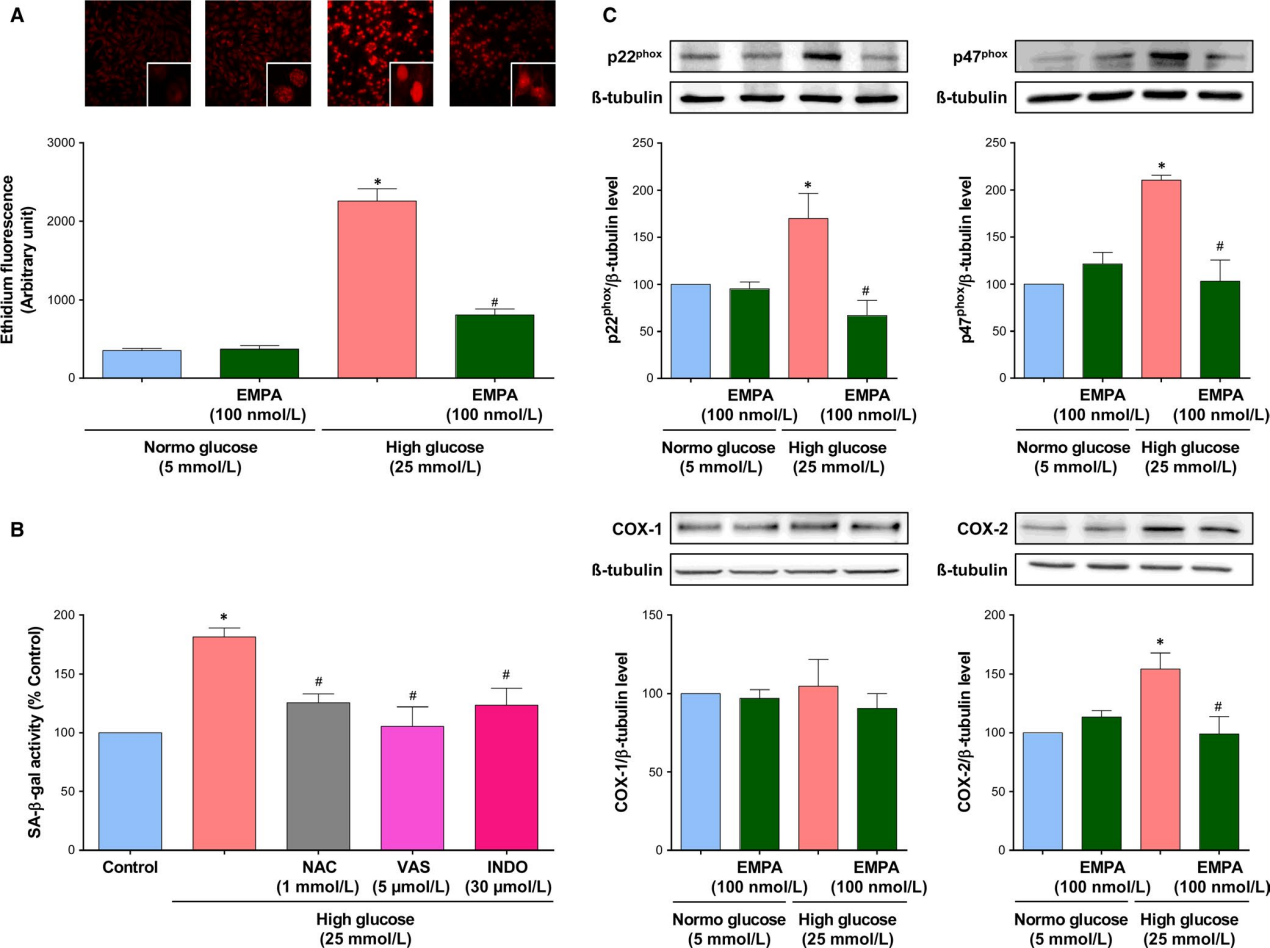
High glucose causes a redox‐sensitive induction of ECs senescence and promotes oxidative stress in ECs involving NADPH oxidase and cyclooxygenases, which is inhibited by empagliflozin. A, ECs are exposed to either normal or high glucose for 24 h in the presence or absence of empagliflozin (100 nmol/L), before dihydroethidium staining. Ethidium fluorescence is determined by confocal microscope. B, ECs are exposed either with N‐acetyl cysteine (NAC, 1 mmol/L), VAS‐2870 (VAS, a NADPH oxidase inhibitor, 5 μmol/L), or Indomethacin (INDO, a COX inhibitor, 30 μmol/L) for 30 min before the addition of high glucose for 96 h, and the subsequent determination of SA‐β‐gal activity by flow cytometry. C, ECs are exposed to normal or a high glucose for 96 h in the presence or absence of empagliflozin (100 nmol/L) before Western blot analysis of the NADPH oxidase subunits p22^phox^, p47^phox^, COX‐1 and COX‐2. Results are shown as representative immunoblots (upper panels) and corresponding cumulative data (lower panels). Data are expressed as mean ± SEM of n = 4‐5. *P <* *0.05 vs. control normal glucose and ^#^ *P *<* *0.05 vs. control high glucose

Since oxidative stress plays a key role in HG‐induced endothelial senescence and dysfunction,^19^ the possibility that empagliflozin affects the oxidative stress activator signal was evaluated. HG markedly increased the level of oxidative stress in ECs at P1 after 24 h, and empagliflozin markedly reduced this effect (Figure 2A). Next, pharmacological tools were used to determine the role of ROS in HG‐induced endothelial senescence and to characterize the enzymatic sources. Pretreatment of ECs with either the anti‐oxidant NAC, the inhibitor of NADPH oxidase (VAS‐2870), or the inhibitor of COXs (indomethacin) significantly reduced the HG‐induced SA‐β‐gal activity (Figure 2B), suggesting the involvement of both NADPH oxidase and COXs in the redox‐sensitive induction of endothelial senescence. Moreover, HG induced the up‐regulation of the expression of the NADPH oxidase subunits p22^phox^ and p47^phox^, and also of COX‐2 but not COX‐1, all of these effects were prevented by empagliflozin (Figure 2C).

### 3.3 High glucose‐induced endothelial dysfunction is prevented by empagliflozin

**Figure 3:**
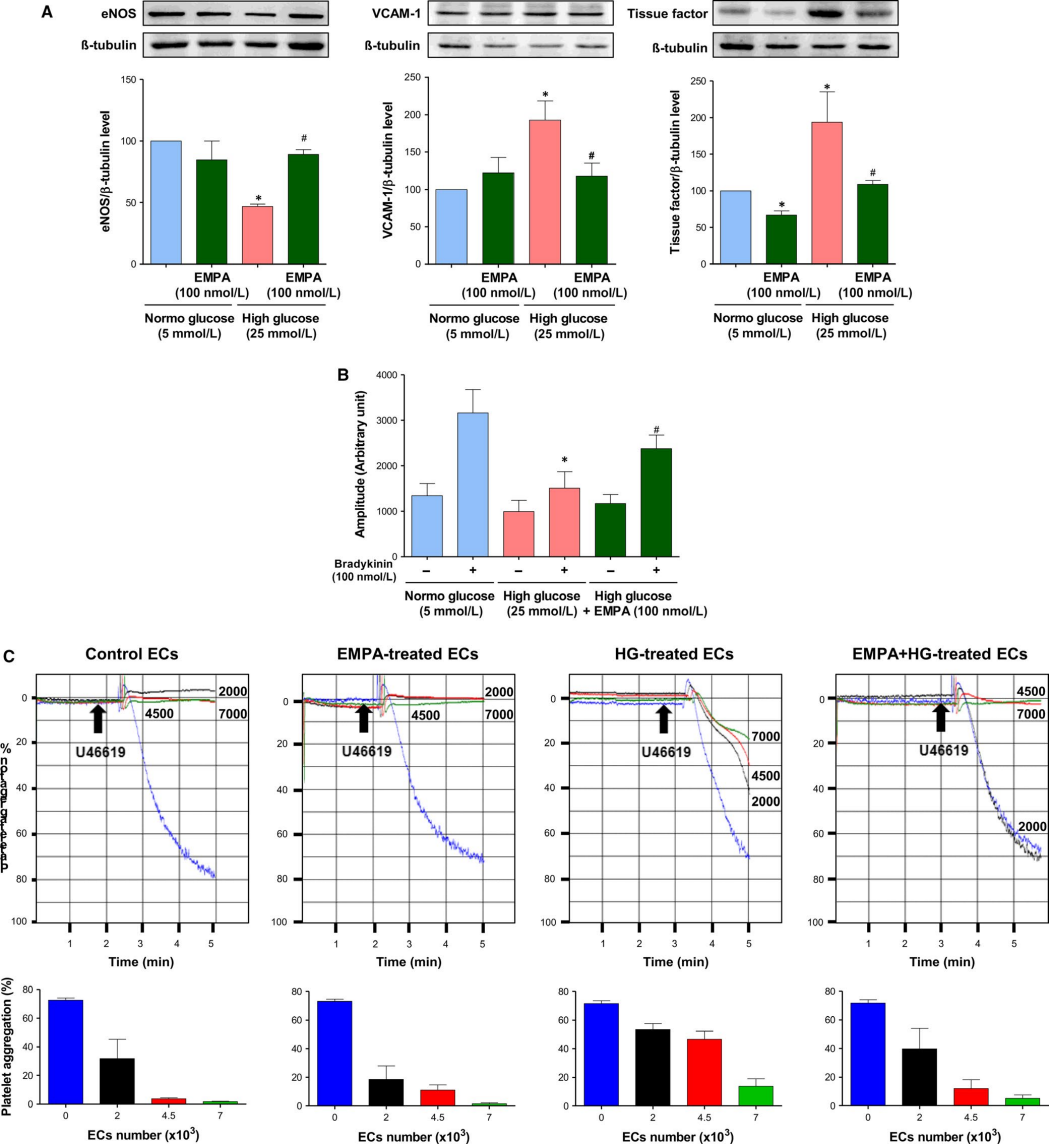
Empagliflozin prevents the high glucose‐induced endothelial dysfunction. ECs are exposed either to normal or high glucose for 96 h in the presence or absence of empagliflozin (100 nmol/L). A, Western blot analysis is performed to determine the expression level of eNOS, VCAM‐1, and tissue factor. Results are shown as representative immunoblots (upper panels) and corresponding cumulative data (lower panels). B, Electron paramagnetic resonance is used to determine the formation of NO under basal conditions and in response to bradykinin (100 nmol/L) for 30 min. C, Platelet aggregation experiments are performed to determine the inhibitory effect of ECs on U46619‐induced platelet aggregation. Representative platelet aggregation curves (upper panels) and corresponding cumulative data (lower panels). Data are expressed as mean ± SEM of n = 3‐4 (A), 3 (B), 4 (C). *P <* *0.05 vs. respective control normal glucose and ^#^ *P *<* *0.05 vs. control high glucose

Since HG and associated oxidative stress are known to promote an impaired eNOS‐derived NO formation and availability, and to activate the expression of pro‐atherothrombotic responses in ECs,^18^, ^19^ experiments have evaluated whether empagliflozin is able to prevent these noxious effects of HG. HG caused a down‐regulation of the expression level of eNOS protein, an up‐regulation of the protein expression level of vascular cell adhesion molecule 1 (VCAM‐1) and tissue factor (Figure 3A), the physiological activator of the coagulation cascade, and a reduced formation of NO in response to bradykinin as evaluated by electron paramagnetic resonance in ECs at P1 (Figure 3B), empagliflozin prevented all of these effects (Figure 3). Since NO is a potent inhibitor of platelet aggregation,^28^ the ability of ECs to inhibit platelet aggregation to U46619, a thromboxane A_2_ analog, was evaluated by adding ECs pre‐treated with either normal glucose or high glucose. As expected, the addition of increasing numbers of ECs at P1 incubated in normal glucose to suspensions of washed human platelets very effectively inhibited platelet aggregation to U46619 (Figure 3C). Since treatment of ECs with the NO synthase inhibitor N^G^‐nitro‐L‐arginine (300 μmol/L) prevented the platelet anti‐aggregatory effect,^32^ it can be attributed to eNOS‐derived NO. In contrast, HG‐treated ECs had a reduced ability to inhibit platelet aggregation whereas empagliflozin partially but significantly restored the anti‐aggregatory effect of the HG‐treated ECs (Figure 3C). The empagliflozin treatment alone did not affect the anti‐aggregatory effect of ECs (Figure 3C).

### 3.4 Role of the local angiotensin system in the HG‐induced ECs senescence

**Figure 4:**
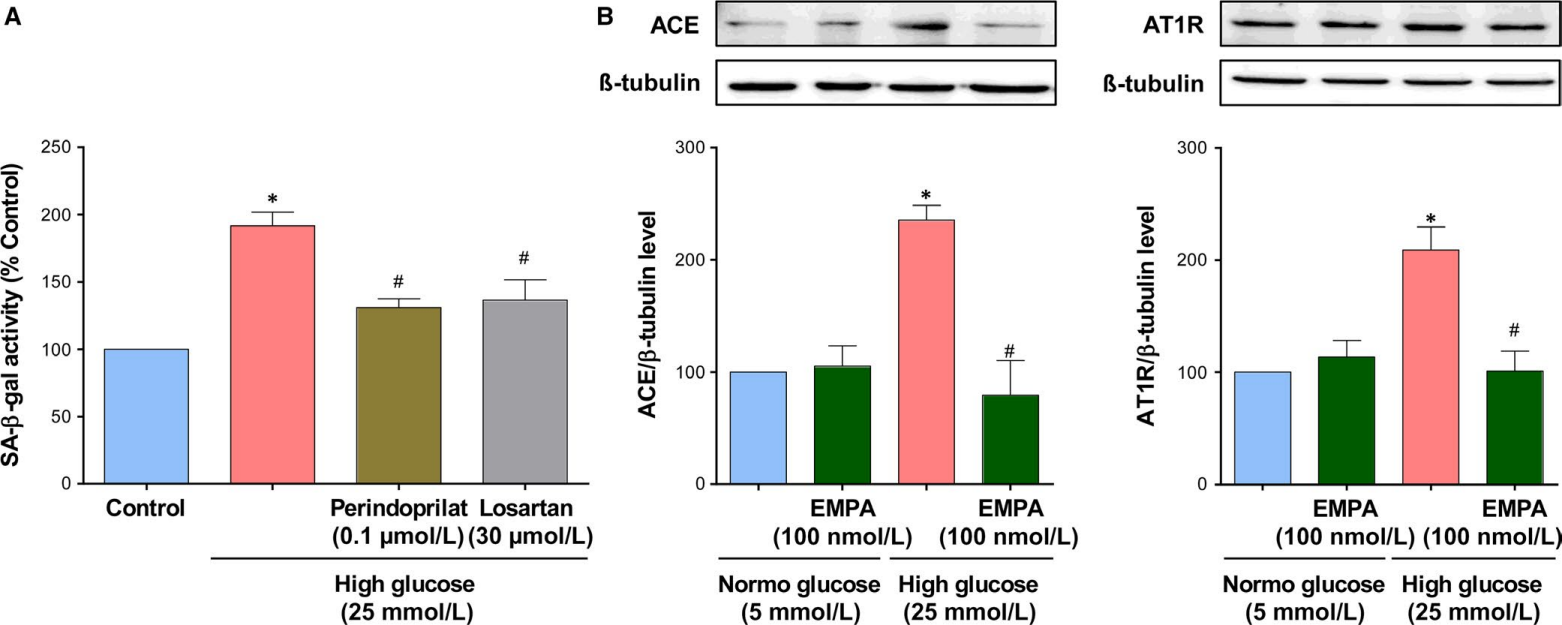
Up‐regulation of the local angiotensin system mediates the high glucose‐induced ECs senescence, an effect prevented by empagliflozin. A, ECs are exposed to either an angiotensin‐converting enzyme inhibitor (Perindoprilat, 0.1 μmol/L) or an AT1R antagonist (Losartan, 30 μmol/L) for 30 min before the addition of high glucose for 96 h, and the subsequent determination of SA‐β‐gal activity by flow cytometry. B, ECs are exposed to normal or a high glucose for 96 h in the presence or absence of empagliflozin (100 nmol/L) before Western blot analysis of angiotensin‐conversion enzyme (ACE), and AT1R. Results are shown as representative immunoblots (upper panels) and corresponding cumulative data (lower panels). Data are expressed as mean ± SEM of n = 3‐4. *P <* *0.05 vs. control normal glucose and ^#^ *P *<* *0.05 vs. control high glucose

Since previous studies have indicated that both AT1 receptor antagonists and inhibitors of angiotensin‐converting enzyme improve the endothelial function in type 2 diabetic patients and in experimental models of diabetes 33, 34, 35 and that Ang II is a strong inducer of endothelial senescence,^36^ experiments were performed to determine the role of the local angiotensin system in HG‐induced endothelial senescence and its modulation by empagliflozin. Both the ACE inhibitor perindoprilat and the AT1R antagonist losartan prevented the HG‐induced increase in SA‐β‐gal activity in ECs (Figure 4A). In addition, HG‐induced endothelial senescence was associated with an increased protein expression level of both ACE and AT1 receptors, both of these effects were abolished by empagliflozin (Figure 4B). Empagliflozin alone affected neither the expression level of ACE nor that of AT1R in ECs (Figure 4 B).

### 3.5 HG promotes SGLT1 and 2‐mediated glucose uptake and expression of SGLT1 and 2 in ECs

**Figure 5:**
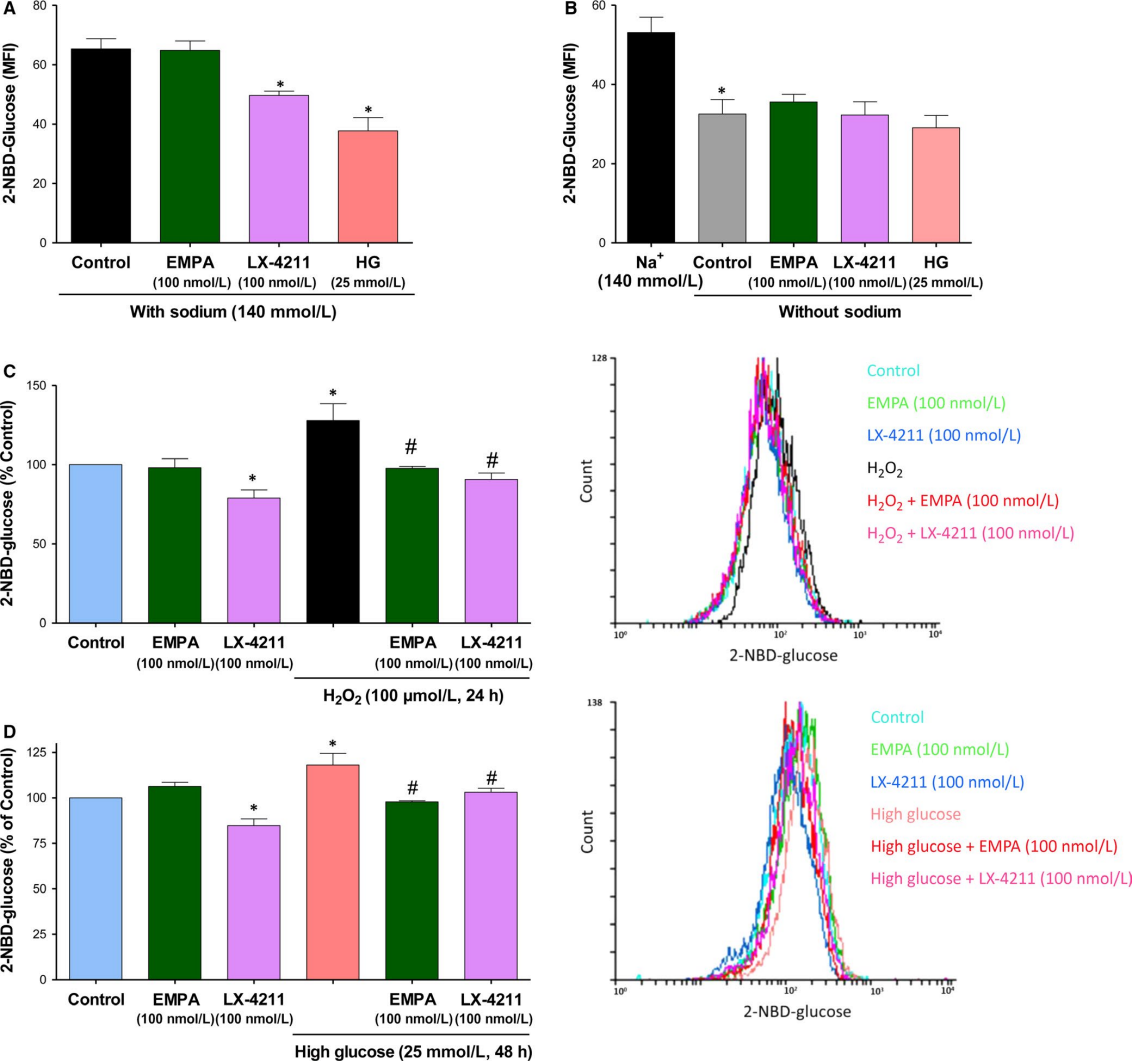
LX‐4211 but not empagliflozin inhibits basal glucose entry into ECs whereas both SGLT inhibitors prevent the increased glucose entry into H_2_O_2_‐ and high glucose‐treated ECs. After a 6‐h incubation period in serum‐free medium without glucose, ECs are incubated with either empagliflozin, LX‐4211 (a dual SGLT1 and 2 inhibitor) or high glucose (25 mmol/L) for 30 min in the presence (A) or absence of sodium replaced by choline chloride (B) before the addition of 2‐NBD‐glucose for 1 h. C,D, ECs are either untreated or exposed to H_2_O_2_ for 24 h, and high glucose for 48 h. After a 6‐h incubation period in serum‐free medium without glucose, ECs are exposed to either empagliflozin or LX‐4211 for 30 min before the addition of 2‐NBD‐glucose for 1 h. Thereafter, the ECs‐associated 2‐NBD‐glucose signal was determined by flow cytometry. Results are shown as cumulative data (left panels) and representative flow cytometry overlay histograms (right panels). Data are expressed as mean ± SEM of n = 3‐5. *P <* *0.05 vs. respective control and ^#^ *P *<* *0.05 vs control H_2_O_2_ or high glucose

**Figure 6:**
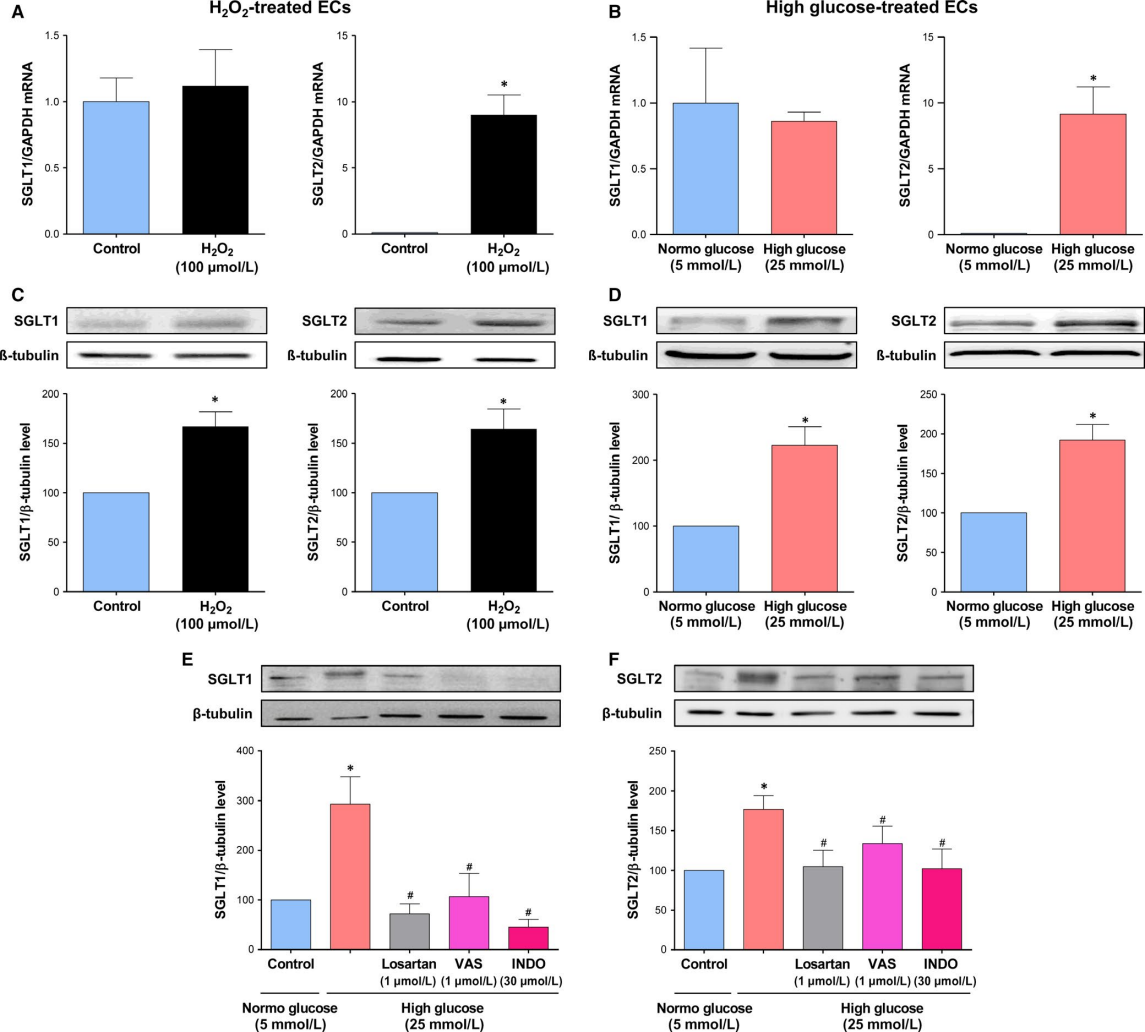
Effect of H_2_O_2_ and high glucose on SGLT1 and SGLT2 mRNA and protein expression in ECs, and role of AT1R, NADPH oxidase and COXs. A,C) ECs are exposed to H_2_O_2_ (100 μmol/L) and, thereafter, the expression level of SGLT1 and 2 mRNA was determined after a 1‐h incubation period by RT‐PCR (A), and SGLT1 and 2 protein after a 24‐h period by Western blot analysis (C). B,D) ECs are exposed to high glucose and, thereafter, the expression level of SGLT1 and 2 mRNA was determined after a 4‐h incubation period by RT‐PCR (B), and SGLT1 and 2 protein after a 96‐h period by Western blot analysis (D). E,F) ECs are exposed to either an AT1R antagonist (Losartan, 1 μmol/L) VAS‐2870 (VAS, a NADPH oxidase inhibitor, 1 μmol/L), or indomethacin (INDO, a COX inhibitor, 30 μmol/L) for 30 min before the addition of high glucose for 96 h and, thereafter, the expression level of SGLT1 and SGLT2 was assessed by Western blot analysis. Results are shown as representative immunoblots (upper panels) and corresponding cumulative data (lower panels). Data are expressed as mean ± SEM of n = 3‐6. *P <* *0.05 vs respective control and ^#^ *P *<* *0.05 vs high glucose

Since previous studies have indicated that native and cultured ECs express SGLT1 mRNA and protein, whereas SGLT2 mRNA was not detectable,^26^, ^27^ experiments were performed to determine if after exposure to HG both SGLTs contribute to glucose uptake into ECs using the fluorescent probe 2‐NBD‐glucose. Under basal conditions, glucose entry into control ECs is competitively inhibited by a high glucose concentration (by about 44.93 ± 4.91%) and reduced by replacement of extracellular Na^+^ by choline chloride by about 38.64 ± 4.87% (Figure 5A,B). Thus, these findings suggest that about 40% of glucose uptake involves Na^+^‐ and glucose‐dependent transport mechanisms. Although empagliflozin did not affect glucose uptake into ECs, LX‐4211 significantly reduced the Na^+^‐ and glucose‐dependent uptake by about 52.88% indicating a major role of SGLT1 in ECs. In addition, H_2_O_2_ increased basal 2‐NBD‐glucose uptake into ECs by about 27% or about 46% when expressed relative to the Na^+^‐ and glucose‐dependent transport mechanism, and high glucose by about 18% or 30% (Figure 5C,D). Both the stimulatory effect of H_2_O_2_ and high glucose on glucose uptake were significantly inhibited by empagliflozin and LX‐4211 suggesting the involvement of both SGLT1 and 2 in glucose transport. Next, the possibility that HG affects the expression of SGLT1 and SGLT2 in ECs was assessed using RT‐PCR and Western blot analysis. Although H_2_O_2_ and HG did not significantly affect SGLT1 mRNA levels observed after 1‐h and 4‐h treatment periods (Ct values were 26.72 ± 0.43 and 26.01 ± 0.48, and 23.65 ± 0.48 and 22.93 ± 0.24), respectively, both treatments significantly increased the SGLT1 protein level by about 1.6 and 2.2‐fold, respectively (Figure 6A‐D). SGLT2 mRNA levels were below the detection level in control ECs even after purification of mRNA (Figure 6A,B) whereas a protein of about 72 kDa was detected by SGLT2 labelling by Western blot analysis (Figure 6C,D). Exposure of ECs to either H_2_O_2_ or HG resulted in the appearance of SGLT2 mRNA levels as assessed following mRNA purification after 1‐h and 4‐h treatment periods (Ct values were 32.57 ± 0.63 and 28.54 ± 0.59, and 34.30 ± 0.85 and 29.42 ± 0.11), respectively, and these effects were associated with a significantly increased SGLT2 protein level by about 1.6 and 1.9‐fold (Figure 6A‐D). In addition, losartan, and also VAS‐2870 and indomethacin prevented the HG‐induced up‐regulation of SGLT1 and SGLT2 protein expression level indicating the involvement of the local angiotensin system, COXs and NADPH oxidase (Figure 6E,F).

### 3.6 HG increases SGLT1 and 2 protein expression levels in native endothelium of coronary artery segments

**Figure 7:**
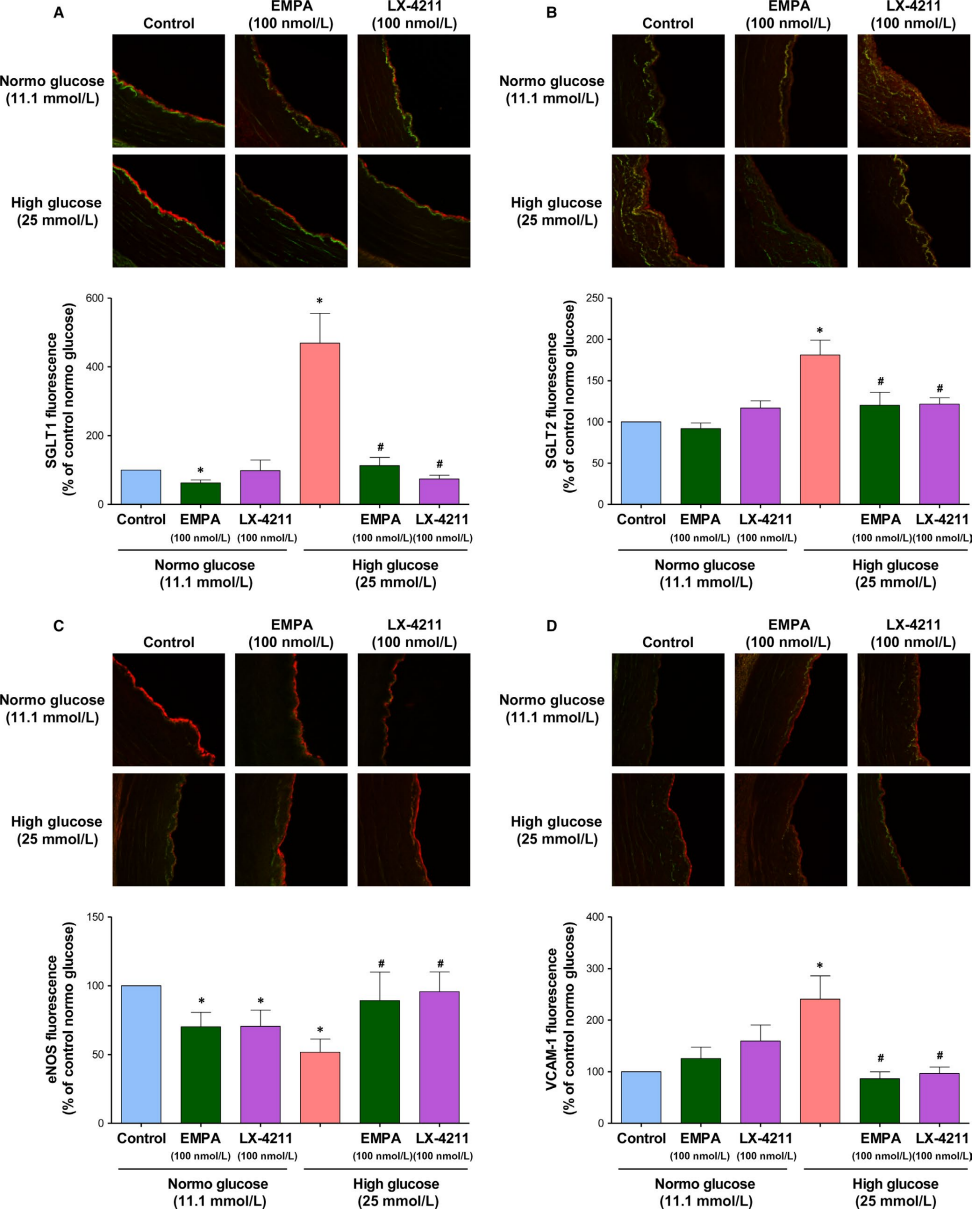
Empagliflozin and LX‐4211 prevent the high glucose‐induced up‐regulation of SGLT1, SGLT2, and VCAM‐1 and down‐regulation of eNOS immunofluorescence signals in the endothelium of coronary artery segments. Porcine coronary artery segments were either untreated, or exposed to empagliflozin (100 nmol/L) or LX‐4211 (100 nmol/L) for 30 min before being incubated in either normo or high glucose containing RPMI for 24 h. Thereafter, segments were embedded in FSC22, frozen in liquid nitrogen and subsequently cryosectioned. Representative confocal immunofluorescence images showing SGLT1 (A), SGLT2 (B), eNOS (C), and VCAM‐1 (D) staining in red and autofluorescence in green (upper panels), and corresponding cumulative data of the endothelial signal (lower panels). Data are expressed as mean ± SEM of n = 3 (A), 4 (B), 8 (C) and 5 (D). *P <* *0.05 vs respective control and ^#^ *P *<* *0.05 vs high glucose. Original magnification, 20x

Since all investigations were performed with cultured ECs, the possibility that HG increases the protein expression level of SGLT1 and 2 in native endothelium, and its potential consequences on responses promoting endothelial dysfunction were assessed using freshly harvested porcine coronary artery segments with endothelium and in the presence of SGLTs inhibitors. A low SGLT1 immunofluorescence signal was detectable predominantly at the luminal surface of the coronary artery whereas that for SGLT2 was barely detectable (Figure 7A,B). Exposure of coronary artery segments to HG for 24 hours resulted in the appearance of a pronounced SGLT1 and a low but consistent SGLT2 signal associated almost exclusively with the endothelium of the coronary artery (Figure 7A,B). Both responses to HG were prevented by empagliflozin and LX‐4211 (Figure 7A,B). Consistent with cultured ECs, HG decreased the eNOS signal and promoted the appearance of a VCAM‐1 signal predominantly in the endothelium, which were both prevented by empagliflozin and LX‐4211 (Figure 7 C,D). Exposure of coronary artery segments to either empagliflozin or LX‐4211 alone affect little baseline signals with the exception of a slight but significant decrease in the low SGLT1 signal by empagliflozin, and a reduction in the eNOS signal by empagliflozin and LX‐4211 (Figure 7).

## 4 DISCUSSION

The major findings of this study indicate that the selective SGLT2 inhibitor empagliflozin very effectively prevented the HG‐induced premature endothelial senescence and dysfunction as characterized by the down‐regulation of the eNOS‐derived NO formation, the expression of pro‐atherosclerotic molecules including tissue factor and VCAM‐1, and a reduced platelet anti‐aggregatory activity. The protective effect of empagliflozin involves its ability to prevent the HG‐induced NADPH oxidase‐ and COXs‐mediated oxidative stress triggering the subsequent activation of the pro‐senescent local angiotensin system and is explained, at least in part, by the up‐regulation of SGLT2 contributing to promote glucose entry into ECs. Importantly, the effect of empagliflozin has been observed within the range of 1‐100 nmol/L, and is in good agreement with the IC_50_ value and drug exposure in clinical practice. Altogether, these observations indicate that SGLT2 appears to be an interesting target to protect the vascular system in diabetes, and possibly also to retard ageing‐ and cardiovascular risk factor‐related endothelial dysfunction.

The recent EMPA‐REG OUTCOME trial has indicated that empagliflozin reduced the risk of major adverse cardiovascular events in type 2 diabetic patients with established cardiovascular disease with a remarkable 38% relative risk reduction in death from cardiovascular causes and a 35% relative risk reduction in hospitalization for heart failure.^22^ Although the mechanisms behind the cardiovascular protective benefits of empagliflozin still remain unclear, several potential mechanisms have been involved such as decreases in arterial stiffness and systolic blood pressure, natriuresis, diuresis and reduced albuminuria, changes in the lipid profile with reduced triglycerides level, reduced sympathetic nervous system activity, improved myocardial energetics by augmenting ketone body oxidation, and small reductions of hyperglycemia, body weight and visceral adiposity.^37^, ^38^ In addition, experimental studies have revealed that empagliflozin improves endothelial dysfunction in streptozotocin‐induced type 1 diabetic rats and in Zucker diabetic fatty rats (type 2 diabetes experimental model 23, 39), and ipragliflozin in streptozotocin‐induced type 1 diabetic mouse.^24^ However, it remains to be clarified whether the protective effect of SGLT2 inhibition on the endothelial function in diabetes is a consequence of the improved cardiorenal function and metabolic parameters including hyperglycemia, and/or also a reduced endothelial glucotoxicity. Indeed, premature endothelial senescence as indicated by an increased SA‐β‐gal positive staining is observed in the aorta of diabetic rats in particular at sites of disturbed blood flow such as bifurcations and branches.^17^, ^18^ Since the expression of the senescence marker p53 in the endothelium promotes blunted endothelium‐dependent relaxations in rat aortic rings,^31^ the induction of endothelial senescence appears to be an early key event triggering endothelial dysfunction. These previous observations in conjunction with the fact that HG is a potent stimulator of premature endothelial senescence in cultured ECs (17, 18; present findings), prompted investigations to clarify the role of SGLT2 in endothelial glucotoxicity.

The present findings indicate that HG‐induced premature endothelial senescence in young ECs at passage 1 was concentration‐dependently prevented by empagliflozin as indicated by reduced SA‐β‐gal activity and reduced activation of the p53/p21 and p16/retinoblastoma protein pathways known to inhibit cell cycle. The characterization of the mechanism underlying the HG‐induced senescence has indicated a determinant role of oxidative stress generated, in part, via the activation of NADPH oxidase and COXs, which is sustained by the up‐regulation of the NADPH oxidase subunits p22^phox^ and p47^phox^ and COX‐2 but not COX‐1. Moreover, since both the ACE inhibitor perindoprilat and the AT1R antagonist losartan abolished the HG‐induced ECs senescence, it implies a pivotal role of the local angiotensin system in the stimulatory effect of HG leading ultimately to endothelial senescence. Furthermore, the up‐regulation of both ACE and AT1R observed in HG‐treated ECs suggests that the local angiotensin system acts in a feed forward mechanism to sustain HG‐induced endothelial senescence. HG‐induced oxidative stress most likely triggers the activation of the local angiotensin system since oxidative stress has been shown to up‐regulate the expression of both ACE and AT1R in endothelial cells and vascular smooth muscle cells.^40^, ^41^ Since SGLT2 inhibition affected neither the redox‐sensitive NADPH oxidase‐, COX‐1‐ and COX‐2‐mediated replicative ECs senescence,^42^, ^43^ nor that induced by H_2_O_2_, one can rule out that empagliflozin acts as an antioxidant. Thus, the protective effect of SGLT2 inhibition is best explained by its ability to prevent the activation of the local angiotensin system subsequent to the effective inhibition of the HG‐induced oxidative stress. In addition, the fact that empagliflozin inhibited only partially the HG‐induced senescence (about 63% at 100 nmol/L) suggests that other mechanisms may also contribute such as glucose entry via SGLT1 and GLUT1 transporters, known to be expressed in ECs,^25^, ^26^, ^44^ hyperosmolarity, and possibly also glucose entry‐independent mechanisms such as the advanced glycation endproducts‐dependent activation of their receptors (RAGEs).

Consistent with previous studies,^18^, ^19^ HG‐induced endothelial senescence promoted pronounced endothelial dysfunction as indicated by the down‐regulation of the eNOS protein level, the reduced bradykinin‐stimulated NO formation, the reduced NO‐mediated platelet anti‐aggregatory capability of ECs, and the up‐regulation of pro‐atherothrombotic markers including the cell adhesion molecule, VCAM‐1, and the physiological activator the coagulation cascade, tissue factor. SGLT2 inhibition very efficiently prevented all components of the HG‐induced endothelial dysfunction, indicating a great potential to protect the endothelium and, hence, the vascular system in diabetes. SGLT2 inhibition has also been shown to normalize the expression of eNOS, components of oxidative stress including Nox1 and Nox2, receptors of advanced glycation endproducts, heme oxygenase‐1 and inflammatory markers in the arterial wall of diabetic rats.^23^, ^24^, ^39^

Since glucose entry into ECs triggers oxidative stress leading to the induction of ECs senescence,^18^, ^19^ the contribution of SGLT2 to glucose entry has been determined using the fluorescent glucose tracer, 2‐NBD‐glucose. The characterization of the glucose uptake has indicated that about 40% involves Na^+^‐ and glucose‐dependent transport mechanisms. Although the dual SGLT1/2 inhibitor LX‐4211 significantly reduced glucose entry into young ECs at passage 1, no such effect was observed in response to SGLT2 inhibition indicating a major contribution of SGLT1 in healthy ECs. These findings are consistent with observations indicating that SGLT1 mRNA and protein are observed in cultured ECs and native endothelium^26,27^ [unpublished data]. Of interest, both the dual SGLT1/2 inhibition as well as the SGLT2 inhibition markedly reduced the pro‐senescent stimulators (HG and H_2_O_2_)‐stimulated glucose entry into ECs suggesting that SGLT2 contributes to glucose entry into pathological ECs. In good agreement with such a concept are the findings that SGLT2 mRNA level was undetectable in control ECs whereas a consistent SGLT2 mRNA level was observed in ECs in response to the pro‐senescent stimulators (HG and H_2_O_2_). Despite undetectable levels of SGLT2 mRNA, a band of about 72 kDa detected by SGLT2 labelling was observed in control ECs, and this signal was significantly higher in HG and H_2_O_2_ stimulated pathological ECs. Similarly, an increased SGLT1 protein expression level was also observed in response to HG and H_2_O_2_. Moreover, the HG‐induced expression of SGLT1 and SGLT2 appears to be mediated via the local angiotensin system most likely subsequent to the NADPH oxidase‐ and COXs‐dependent oxidative stress. Of interest, since HG caused a down‐regulation of the predominant GLUT in ECs, the ubiquitous GLUT‐1,^44^ the up‐regulation of SGLT1 and 2 transport may contribute to promote endothelial dysfunction in response to HG.

The present findings showing a dissociation between mRNA and protein levels of both SGLT1 and SGLT2 in H_2_O_2_‐ and HG‐treated ECs is consistent with previous observations. Indeed, the BTBR ob/ob type 2 diabetic mice is characterized by about a 50% reduction in the SGLT1 mRNA level associated with a 2‐fold higher SGLT1 protein level in the renal cortex.^45^ Moreover, the injection of a glucose solution directly into the intestinal lumen of ruminant sheep for 4 days resulted in an increased SGLT1 mRNA level by about 2‐fold in the proximal intestine whereas SGLT1 protein and activity levels were increased by about 60‐90‐fold.^46^ Thus, these findings indicate that the principal level of SGLT1 regulation by luminal sugar is translational or post‐translational. In addition, a 2.5‐fold higher SGLT2 protein level has been observed in the renal cortex of adult female compared to male rats despite a similar expression level of SGLT2 mRNA.^47^ Thus, these previous findings in conjunction with the present ones indicate that changes in SGLT1 and SGLT2 mRNA levels are not associated with corresponding changes in the respective protein levels demonstrating the involvement of complex regulatory mechanisms that still remain to be clarified.

Moreover, the present findings provide also evidence that HG is able to up‐regulate markedly SGLT1 and, also to some extent, SGLT2, almost exclusively in the native endothelium of porcine coronary arteries, and that this effect is associated with a down‐regulation of eNOS and an up‐regulation of VCAM‐1 in the endothelium. Since both empagliflozin and LX‐4211 very effectively prevented all stimulatory effects of HG on the endothelium, both SGLTs appear to play a key role.

In conclusion, the major novel findings of this study indicate that SGLT2, as well as SGLT1, is expressed in cultured and native ECs under pathological conditions including hyperglycemic conditions and oxidative stress. They further indicate that SGLT2 appears to contribute to excessive glucose entry promoting endothelial senescence and dysfunction, in part, via the local angiotensin system acting in a feed forward mechanism to sustain oxidative stress. Since SGLT2 expression is up‐regulated by oxidative stress in ECs, SGLT2 appears to be an interesting target to prevent the induction of endothelial senescence and dysfunction and, hence, to retard premature vascular ageing.

## CONFLICT OF INTEREST

This work was supported by an unrestricted research grant from Boehringer Ingelheim Pharma GmbH & Co. KG, Biberach, Germany.

## AUTHOR CONTRIBUTION

SK‐B, EB, NI‐K, S‐HP, LA, MA: performed the research and analyzed the data. SK‐B, CA, LK, EM, FT, VS‐K: designed the research study; wrote the paper.
